# Combination of 7-*O*-geranylquercetin and microRNA-451 enhances antitumor effect of Adriamycin by reserving P-gp-mediated drug resistance in breast cancer

**DOI:** 10.18632/aging.204287

**Published:** 2022-09-14

**Authors:** Yuling Chen, Xiaohong Li, Lei Shi, Pengfei Ma, Wei Wang, Nan Wu, Youlin Gan, Xu Han, Shanshan Huang, Xiaohui Kang, Shuxin Liu, Yuhong Zhen

**Affiliations:** 1 College of Pharmacy, Dalian Medical University, Dalian 116044, China; 2 Affiliated Dalian Friendship Hospital of Dalian Medical University, Dalian 116001, China; 3 The First Affiliated Hospital of Dalian Medical University, Dalian 116023, China; 4 The Second Affiliated Hospital of Dalian Medical University, Dalian 116011, China; 5 Affiliated Dalian Municipal Central Hospital of Dalian Medical University, Dalian 116033, China; 6 Dalian Key Laboratory of Intelligent Blood Purification, Dalian 116033, China

**Keywords:** 7-*O*-geranylquercetin, microRNA-451, P-glycoprotein, drug resistance, Adriamycin

## Abstract

Although there are a lot of chemical drugs to treat breast cancer, increasing drug resistance of cancer cells has strongly hindered the effectiveness of chemotherapy. ATP-binding cassette transporters represented by P-glycoprotein (P-gp), multidrug resistance associated protein 1 (MRP1) and breast cancer resistance protein (BCRP) play an important role in drug resistance. This study aims to investigate the effect of 7-*O*-geranylquercetin (GQ) combining microRNA-451(miR-451) on reversing drug resistance of breast cancer and reveal the mechanism related to P-gp. Real-time RT-PCR and western blot assays showed that miR-326, miR-328, miR-451 and miR-155 inhibitor down-regulated the expression of genes MRP1, BCRP, MDR1 and the corresponding proteins MRP1, BCRP, P-gp, respectively. Cell counting kit-8 (CCK-8) assay indicated that these miRNAs reversed the resistance of MCF-7/ADR cells to Adriamycin (ADR), and miR-451 showed the greatest reversal effect. Combination of GQ and miR-451 enhanced the inhibitory effects of ADR on the proliferation and migration of MCF-7/ADR cells, and attenuated the expression of MDR1 and P-gp in MCF-7/ADR cells. A xenograft tumor model was used to show that GQ and miR-451 amplified the antitumor effect of ADR in nude mice, while western blot and immunohistochemical assays revealed the decreased expression of P-gp in tumor tissues. These results suggest that GQ and miR-451 have synergistic effect on reversing drug resistance through reducing the expression of MDR1 and P-gp in breast cancer MCF-7/ADR cells.

## INTRODUCTION

Breast cancer has become the most common female malignant tumor, with the morbidity and mortality both ranking first [1]. Multi-drug resistance (MDR) of cancer cells to chemotherapeutics is the main reason for the low cure rate in tumor therapy [2]. ATP-binding cassette (ABC) transporters represented by multidrug resistance associated protein 1 (MRP1), P-glycoprotein (P-gp) and breast cancer resistance protein (BCRP) play an important role in MDR [2–4]. So developing safe and effective inhibitors of ABC transporters to overcome MDR has become top priority [5].

Quercetin is a natural flavonoid compound that has ability to attenuate Adriamycin (ADR)-resistance induced by ABC transporters [6]. But its clinical application is restricted by the lower solubility [7]. Quercetin has to be administrated at high doses to reach the concentration for reversion of drug resistance, which may cause cytotoxicity to normal cells [8]. 7-*O*-Geranylquercetin (GQ) is a liposoluble modified product of quercetin in our laboratory [9]. We have found that high concentration of GQ restrained the growth and promoted the apoptosis of human gastric cancer, breast cancer and lung cancer cells [9–11]. GQ could reverse ADR-resistance of breast cancer cells at low concentration, and showed stronger reversal effect than quercetin, indicating that it is a promising MDR reverser [12].

Many studies have revealed that occurrence, metastasis and MDR of tumors were closely related to the dysfunction of microRNAs (miRNAs) and confirmed that some miRNAs were involved in drug resistance by regulating ABC transporters [13]. So, these miRNAs are potential targets for drug-resistance reversion in tumor therapy. Most of these miRNAs reduce drug resistance by negatively regulating the expression of ABC transporters in tumor cells. For example, microRNA-451 (miR-451), microRNA-326 (miR-326) and microRNA-328 (miR-328) could amplify the drug sensitivity of tumor cells by down-regulating the expression of MDR1, MRP1 and BCRP genes, and consequently inhibiting the expression of P-gp [14], MRP1 and BCRP proteins [15], respectively. In contrast, a few microRNAs increase the expression of ABC transport proteins and then induce drug resistance in cancer cells, such as microRNA-155 (miR-155) [16].

Studies have shown that siRNA and small molecule agents, such as doxorubicin, carboplatin and paclitaxel, had synergistic effect on inhibiting tumor proliferation, metastasis and drug resistance [17, 18]. However, the cooperation of small molecule compounds with miRNAs in reversing drug resistance is seldom reported so far. This study explored the regulating effect of miRNAs on ABC transporters and further probed into the synergetic action of GQ and miR-451 on reversing P-gp-mediated drug resistance in MCF-7/ADR (ADR-resistant) cells, aiming to provide new ideas for the clinical therapy of MDR tumors.

## RESULTS

### MiRNAs reversed ADR-resistance in MCF-7/ADR cells

**Figure 1:**
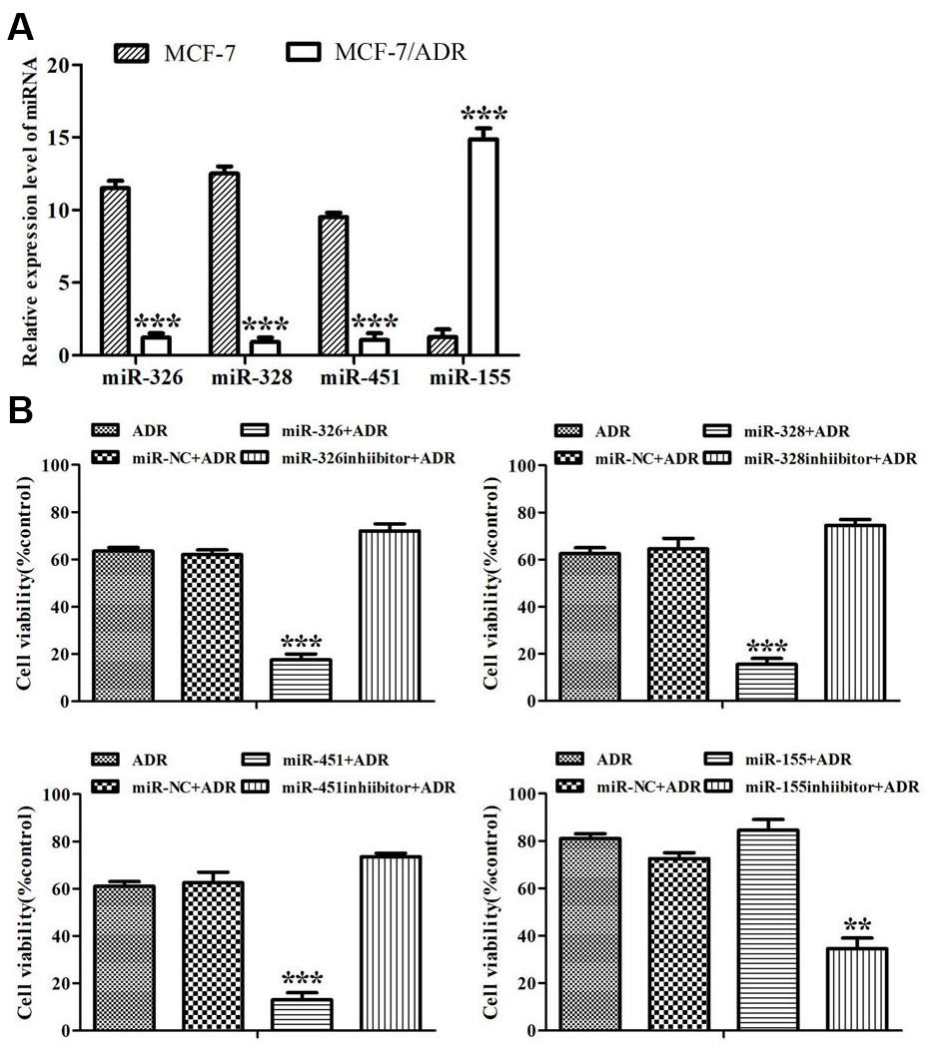
**MiRNAs reversed ADR-resistance in MCF-7/ADR cells.** (**A**) Expression of miR-326, miR-328, miR-451 and miR-155 in MCF-7 and MCF-7/ADR cells were detected by Real-time RT-PCR. ^***^P < 0.001, comparing with MCF-7 cells. (**B**) MCF-7/ADR cells and MCF-7/ADR cells transfected with miRNAs were treated with ADR (43 μM) for 48 h. Cell viability was measured by CCK-8 assay. All data represent the means ± SD of three independent experiments. ^**^P < 0.01, ^***^P < 0.001, comparing with non-transfection group.

It was shown that miR-326, miR-328 and miR-451 expressed at low levels in ADR-resistant cells while miR-155 expressed at a high level. On the contrary, miR-326, miR-328 and miR-451 showed high levels in MCF-7 cells, while miR-155 showed a low level (Figure 1A). This demonstrated that these four miRNAs might be related to ADR-resistance in breast cancer cells.

When ADR-resistant cells were subjected to ADR for 48 h, the proliferation rate of miR-NC-transfected cells was similar to that of untransfected cells, but the proliferation rates of the cells transfected with miR-326, miR-328, miR-451 and the inhibitor of miR-155 dropped from 60-80% to 19%, 18%, 17% and 27%, respectively (Figure 1B). The results showed that these miRNAs reversed drug resistance in ADR-resistant cells.

We also detected the reversal folds of miRNAs to ADR-resistance. IC_50_ values of ADR to MCF-7 and ADR-resistant cells were respectively 5.73 μM and 61.51 μM, and the resistance fold was 10.73. Drug resistance reversal folds of miR-326, miR-328, miR-451 and the inhibitor of miR-155 to ADR-resistant cells were 3.15, 3.0, 3.76, 2.02, respectively (Table 1). These results indicated that miR-451, miR-326, miR-328 and the inhibitor of miR-155 could reverse ADR-resistance, and miR-451 showed the highest reversal effect.

**Table 1:** Reversal effect of miRNAs on ADR-resistance in MCF-7/ADR cells.

	**MCF-7**	**MCF-7/ADR**	**MCF-7/ ADR**				
			**miR-326**	**miR-328**	**miR-451**	**miR-155 inhibitor**	**miR-451 +GQ**
ADR IC_50_ (μM)	5.73±0.12	61.51±0.86	19.87±0.20	20.43±0.32	16.34±0.13	30.41±0.19	2.93±0.42
Resistance fold	—	10.73	3.46^***^	3.57^***^	2.85^***^	5.31^***^	0.51^***^
Reversal fold	—	—	3.15	3.0	3.76	2.02	21.04

MCF-7 cells, MCF-7/ADR cells and MCF-7/ADR cells transfected with miRNAs were treated with ADR (172, 86, 43, 21.5, 10.75, 5.33, 2.67, 1.33, 0.67 and 0 μM) for 48 h. In addition, miR-451-transfected MCF-7/ADR cells were treated with ADR and GQ (10 μM) for 48 h. Cell viability was detected by CCK-8 assay, and the IC_50_ values were calculated. All data represent the means ± SD of three independent experiments. ^***^P < 0.001, comparing with MCF-7/ADR cells without transfection.

### MiRNAs regulated the expression of MRP1, BCRP, MDR1 genes and MRP1, BCRP, P-gp proteins

**Figure 2:**
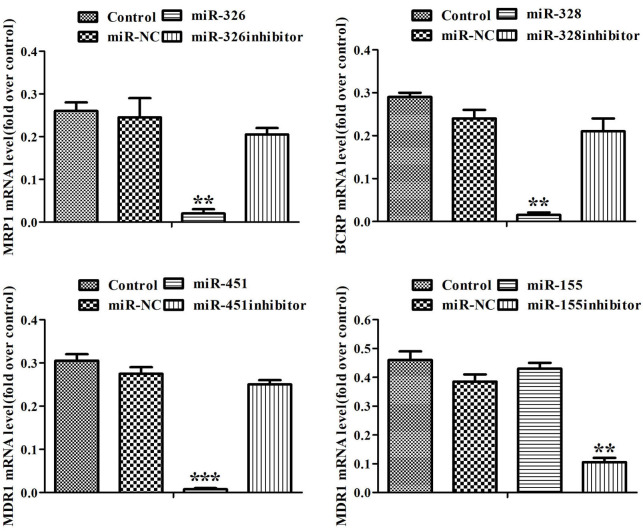
**MiRNAs regulated the expression of MRP1, BCRP, MDR1 genes.** The expression levels of MRP1, BCRP and MDR1 in MCF-7/ADR cells and MCF-7/ADR cells transfected with miRNAs were detected by Real-time RT-PCR. All data represent the means ± SD of three independent experiments. ^**^P < 0.01, ^***^P < 0.001, comparing with control group.

**Figure 3:**
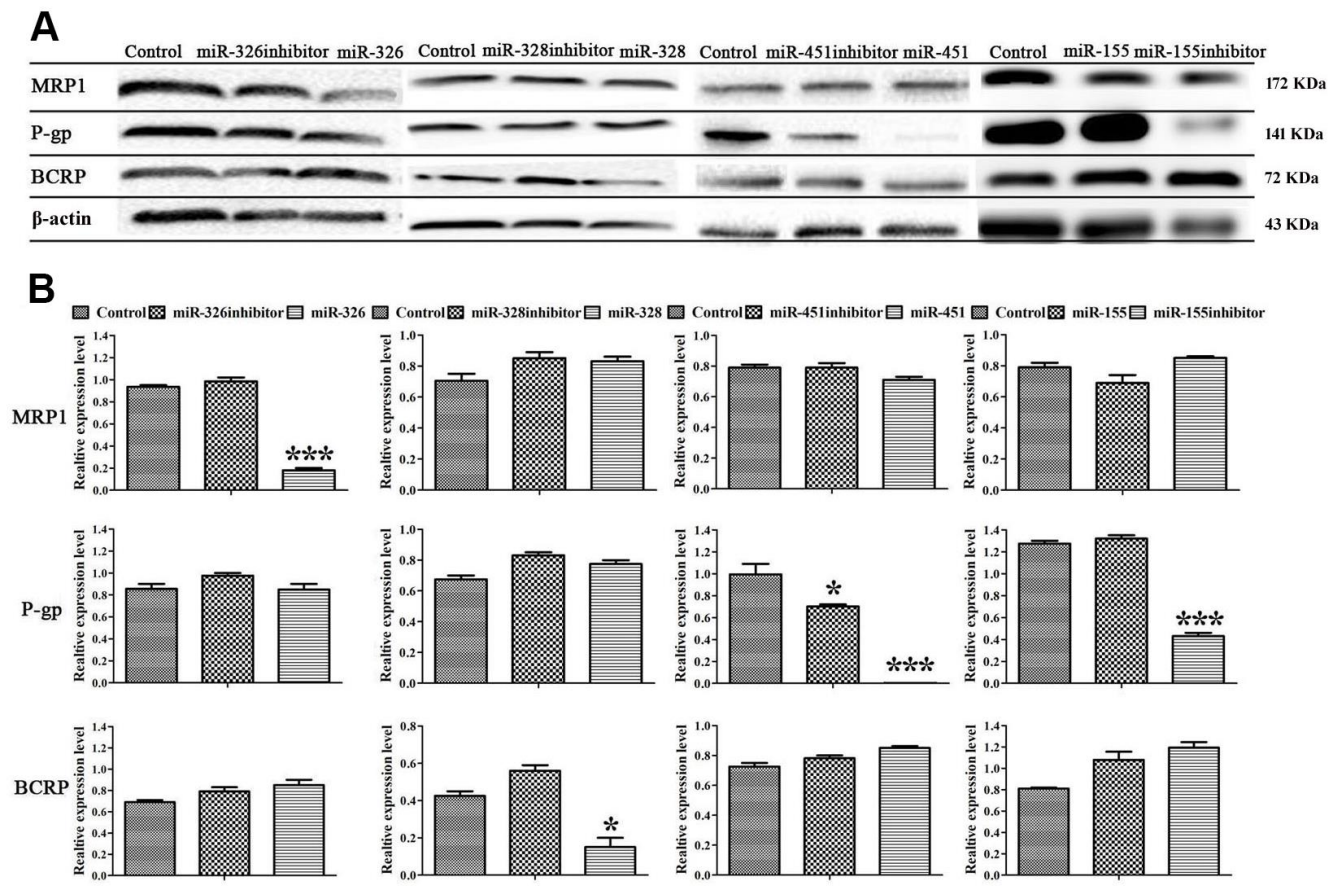
**MiRNAs regulated the expression of MRP1, BCRP and P-gp proteins.** (**A**) Western blot was conducted to detect the expression of MRP1, BCRP, P-gp and β-actin in MCF-7/ADR cells and MCF-7/ADR cells transfected with miRNAs. (**B**) Quantitative analysis of the results in (**A**). All data represent the means ± SD of three independent experiments. ^*^P < 0.05, ^***^P < 0.001, comparing with control group.

To disclose the mechanism of miRNAs reversing drug resistance, the expression levels of MRP1, BCRP, MDR1 genes and MRP1, BCRP, P-gp proteins in ADR-resistant cells were tested after transfection of miRNAs. Real-time RT-PCR assay showed that miR-326 and miR-328 down-regulated the expression levels of MRP1, BCRP respectively, miR-451 and the inhibitor of miR-155 down-regulated the expression level of MDR1 (Figure 2). Western blot showed that miR-326 and miR-328 suppressed the expression of MRP1 and BCRP, while miR-451 and the inhibitor of miR-155 suppressed the expression of P-gp. MiR-451 showed the strongest inhibition effect on gene and protein expression (Figure 3). Above results revealed that miRNAs attenuated the expression of transporters MRP1, BCRP and P-gp by suppressing the expression of MRP1, BCRP and MDR1 genes in ADR-resistant cells.

### Combination of GQ and miR-451 enhanced the inhibitory action of ADR on proliferation and migration of MCF-7/ADR cells

With transfection of miR-451 into ADR-resistant cells, the IC_50_ of ADR decreased from 61.51 μM to 16.34 μM, and then to 2.93 μM with additional treatment of GQ. Drug resistance reversal fold of miR-451 to ADR was 3.76, while that of GQ combining miR-451 was 21.04 (Table 1), indicating that combination of GQ and miRNA-451 more effectively reversed ADR-resistance in MCF-7/ADR cells.

**Figure 4:**
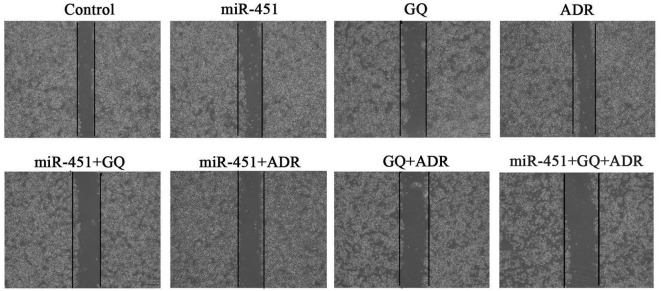
**Effect of GQ and miR-451 on the migration inhibition of ADR to MCF-7/ADR cells.** MCF-7/ADR cells or miR-451-transfected MCF-7/ADR cells were seeded on 6-well plates with monolayer and divided on average with a UV-sterilized ruler and pipette tips. Then the cells were treated with GQ (10 μM), ADR (43 μM) or their combination for 48 h and photographed using an inverted microscope.

In scratch experiment, ADR, GQ, miR-451 alone or GQ combining miR-451 showed slight inhibition effect on the migration of ADR-resistant cells. The inhibitory action of ADR was enhanced via being combined with miR-451 or GQ, and ADR combined with GQ and miR-451 showed the strongest inhibition effect on cell migration (Figure 4). These data demonstrated that GQ combining with miR-451 exerted synergistic effect on reversing ADR-resistance in MCF-7/ADR cells.

### Combination of GQ and miR-451 inhibited the expression of MDR1 and P-gp in MCF-7/ADR cells

**Figure 5:**
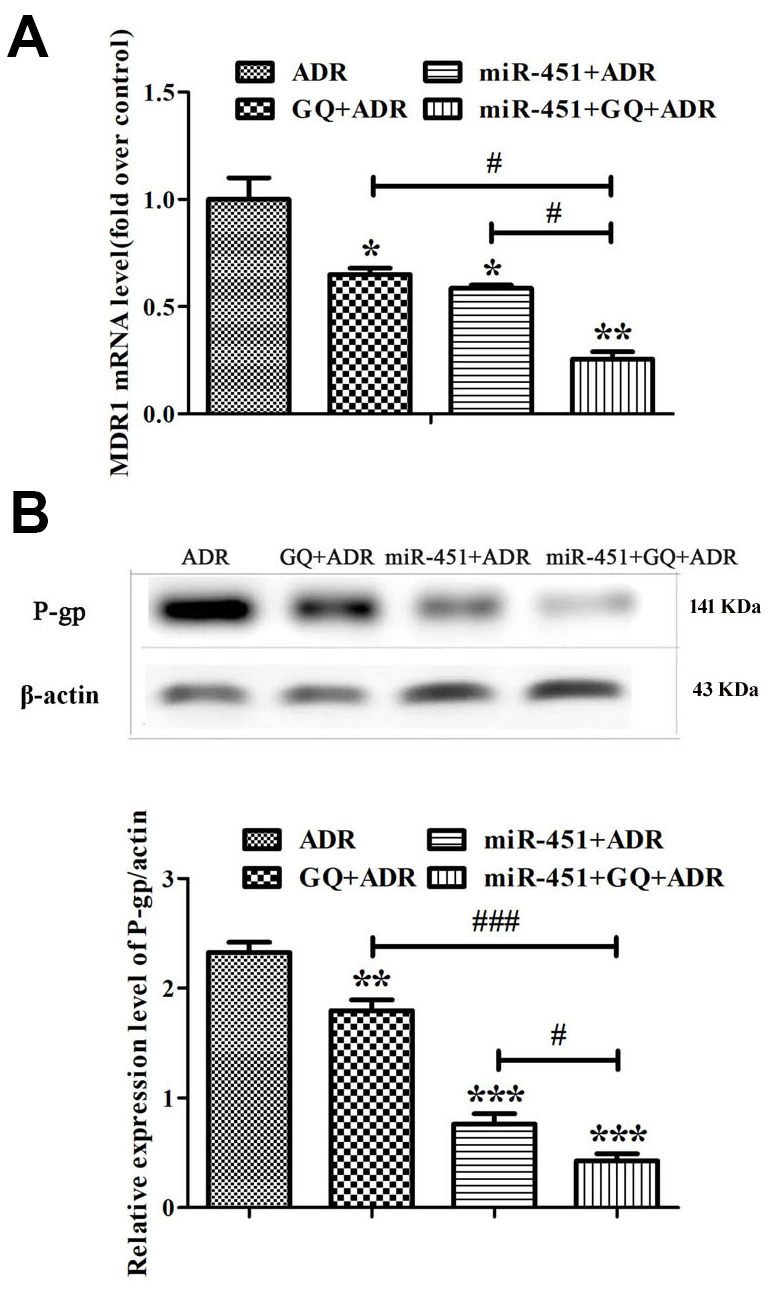
**Combination of GQ and miR-451 inhibited the expression of MDR1 and P-gp.** MCF-7/ADR cells and miR-451-transfected MCF-7/ADR cells were subjected to ADR (43 μM) or ADR (43 μM) combining GQ (10 μM) for 48 h. (**A**) Expression levels of MDR1 were detected by Real-time RT-PCR. The MCF-7/ADR cells only treated with ADR were regarded as control. (**B**) Expression levels of P-gp were detected by western blot. All data represent the means ± SD of three independent experiments. ^*^P < 0.05, ^**^P < 0.01, ^***^P < 0.001, comparing with ADR group; ^#^*P* < 0.05, ^###^*P* < 0.001, comparing with miR-451, GQ and ADR combination group.

Real-time RT-PCR showed that both miR-451 and GQ significantly inhibited MDR1 expression in ADR-treated MCF-7/ADR cells, and the inhibitory action of GQ combining miR-451 was more potent than that of GQ or miR-451 (Figure 5A). Western blot showed that miR-451 and GQ markedly inhibited P-gp expression in ADR-treated MCF-7/ADR cells, the inhibitory action of GQ combining miR-451 was stronger than that of GQ or miR-451 alone (Figure 5B). These indicated that GQ combining miR-451 inhibited MDR1 and P-gp expression, and had a stronger capability of reversing drug resistance as compared to either of them.

### Combination of GQ and miR-451 enhanced the inhibition effect of ADR on tumor growth

**Figure 6:**
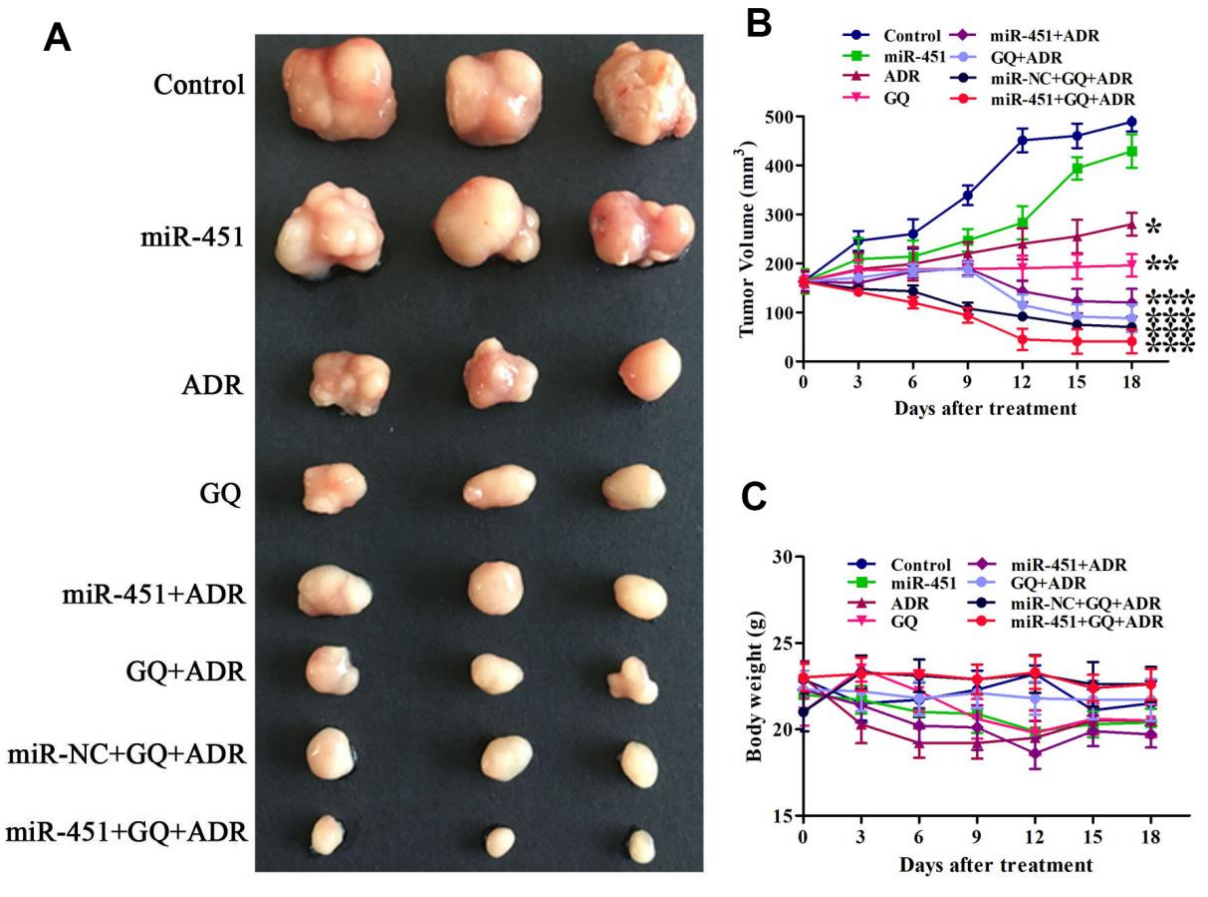
**GQ and miR-451 enhanced the inhibitory effect of ADR on tumor growth in mice.** Tumor-bearing mice were treated with ADR (3 mg/kg) or the complex of miR-451 (0.55 mg/kg) and liposome CDO14 (1.5 mg/kg) via tail vein injection, or treated with GQ (18 mg/kg) by gavage for 6 times at an interval of 3 days. (**A**) Tumors removed from the mice after treatment. (**B**) Volume of tumor in mice during the treatment. (**C**) Body weight of mice during the treatment. All data represent the means ± SD, n=3. ^*^P < 0.05, ^**^P < 0.01, ^***^P < 0.001, comparing with control group.

After proving the drug resistance reversing effect of GQ combining miR-451 in ADR-resistant cells, we then evaluated their synergetic drug resistance reversing effect on xenograft in mice. The tumor in mice treated only with GQ, ADR or miR-451 continuously grew during the treatment, whereas the tumor growth rate in mice treated with GQ or ADR was slower than that in miR-451-transfected mice or control mice. The tumor volume of mice treated with ADR combined with GQ or/and miR-451 reduced obviously. The tumor growth inhibition effect of ADR combined with GQ and miR-451 was stronger than that of ADR combined with GQ or miR-451 (Figure 6A, 6B). The body weight of mice treated with ADR or ADR combined with miR-451 showed a slight downward trend while that of mice in other groups had no significant change during the treatment, revealing that GQ, miR-451 and liposome CDO14 did not induce apparent toxicity in mice (Figure 6C). All of these suggested that GQ and miR-451 augmented tumor suppressive action of ADR *in vivo*.

### Combination of GQ and miR-451 depressed P-gp expression in tumor tissues

**Figure 7:**
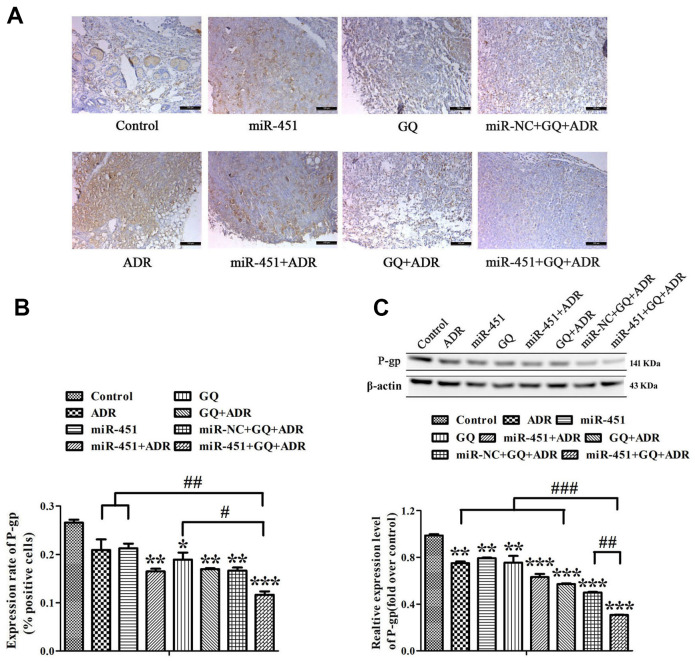
**Effect of GQ and miRNA on the expression of P-gp in tumor tissues.** (**A**) P-gp expression levels in tumor tissues were detected by IHC assay, the bar indicated 100 μm; (**B**) Quantitative analysis of the results in (**A**); (**C**) P-gp expression levels in tumor tissues were detected by western blot. All data represent the means ± SD of three independent experiments. ^*^P < 0.05, ^**^P < 0.01, ^***^P < 0.001, comparing with control group. ^#^*P* < 0.05, ^##^*P* < 0.01, ^###^*P* < 0.001, comparing with miR-451, GQ and ADR combination group.

Immunohistochemistry (IHC) assay showed that P-gp-positive area in tumor tissues of mice subjected to GQ, ADR combined with GQ or/and miR-451 was obviously less than that in control group, and P-gp-positive area was the least in the group treated with ADR combined with GQ and miR-451 (Figure 7A, 7B). Western blot assay meanwhile demonstrated that the expression level of P-gp was the lowest in the tumors of ADR combined with GQ and miR-451 treatment group (Figure 7C). So, it was confirmed that GQ and miR-451 reversed ADR-resistance in MCF-7/ADR cells by depressing P-gp expression, thereby increasing tumor suppression effect.

## DISCUSSION

Up to now, three generations of P-gp inhibitors are in research and development, but few of them are satisfactory for clinical application [12]. Our previous study had shown that GQ could reverse ADR-resistance in MCF-7/ADR cells [12]. This study proved that miRNAs also had reverse effect, and GQ combining miR-451 showed stronger reverse effect than either of them.

MiRNAs regulate the expression of target genes through causing their degradation or translation inhibition, thereby changing the expression level of target proteins [19]. Studies showed that miRNAs were often abnormally expressed in tumor cells and associated with tumorigenesis, metastasis, invasion and drug resistance [20–23]. Cancer-related miRNAs are divided into cancer promoter and suppressor. The endogenous level of miRNAs acting as tumor suppressor, such as miR-451, was lower in tumor tissues than in normal parts [24]. In contrast, the endogenous level of miRNAs acting as cancer promoter, such as miR-155, was higher in tumor tissues than in normal parts [25].

Increasing evidences suggested that miRNA-derived strategies had a great prospect in cancer therapy [26]. In most instances, tumors are responsive to the initial treatment, but soon be in aggressive form because of MDR [27]. Accordingly, exploring safe and effective MDR-reversing targets such as miRNAs is very important and urgent for cancer treatment [28]. MiRNAs can modulate drug resistance by participating in the expression of cell membrane proteins [29] and apoptotic genes [30]. MRP, BCRP and P-gp belong to the typical ABC transporter family [4], a group of transmembrane proteins, which can pump drugs out of tumor cells. Over-expression of MRP, BCRP and P-gp, as a classical drug resistance mechanism, can increase the efflux capacity of cells to chemotherapeutic drugs, making drug accumulation decrease or even disappear and then reducing the toxicity to cancer cells [31]. Regulating the expression of ABC transporters with miRNAs to restore drug-susceptibility of cancer cells is instrumental in cancer therapy. Shi et al. revealed that miRNA-29a could reverse drug resistance mediated by P-gp in colon cancer cells [28]. Here we demonstrated that miR-326, miR-328 and miR-451 were expressed at low levels, while miR-155 was expressed at a high level in drug-resistant cells. We then proved that miR-326, miR-328, miR-451 and the inhibitor of miR-155 reversed ADR-resistance via down-regulating the expression of MRP1, BCRP and MDR1, then respectively attenuating the expression of MRP1, BCRP, P-gp in ADR-resistant cells. MiR-451 showed the strongest inhibition and was selected to combine with GQ to evaluate the synergistic reversal effect of miRNAs and small molecule agents on MDR.

In our previous studies, GQ showed good inhibitory effect to various cancer cells at concentrations higher than 10 μM [9–11]. In order to avoid the influence of its antitumor action, GQ was used at 10 μM in MDR reversing study. We had reported that GQ cut down the ADR efflux by suppressing P-gp expression [12]. We also found in molecular docking model that GQ bond to P-gp through hydrophobic interaction and hydrogen bonds [12]. So, GQ could not only control the expression of P-gp but also directly interact with it, resulting in MDR reverse effect. This study demonstrated that miR-451 attenuated P-gp expression by down-regulating MDR1 gene. We further concluded that GQ and miR-451 synergistically reversed ADR-resistance by binding with P-gp and down-regulating its expression.

The present study indicated that GQ and miR-451 reinforced the antitumor activity of ADR in mice. The body weight of mice did not change obviously during the treatment, because GQ and miR-451 promoted ADR accumulation in tumor cells and reduced ADR distribution in normal tissues, thereby attenuating its toxicity. The results of IHC and western blot indicated that P-gp level in tumor tissues of mice treated with ADR combined with GQ or miR-451 was significantly down-regulated, which was similar to that in ADR-resistant cells. Tumor suppressive effect of ADR combined with GQ and miR-451 simultaneously was stronger than being combined with GQ or miR-451 alone.

Although our research indicated that GQ combining miR-451 had better ADR-resistance reversing effect than using a single drug, inconvenience during therapy might be aroused from the difference in administration routes of GQ and miR-451. Liang et al. reported that exosomes co-loaded with miR-21 inhibitor and anticancer drug 5-fluorouracil could be considered as a potential strategy to reverse 5-fluorouracil resistance in colorectal cancer [32]. Rui et al. also found that co-delivery of miR-21 inhibitor and doxorubicin prodrug by mimetic lipoprotein nanoparticles could be a promising application in reversing drug resistance in cancer cells [33]. Though co-delivery of drug and gene is attracting more and more attention, the strategy implementation is limited by the complexity of drug administration.

In this study, miRNAs were transfected by liposome CDO14 which was a peptide cationic liposome developed in our lab and proved to be highly effective and low toxic for gene transfection [34, 35]. We also found that GQ and siRNA-IGF-1R co-delivered by CDO14 had synergistic action on human non-small cell lung cancer [36]. In the future, we will try to encapsulate ADR and GQ in a cationic nanocarrier such as liposome CDO14, and co-deliver miRNA to reduce the side-effect of ADR and meanwhile achieve the purpose of one-time administration.

## CONCLUSIONS

The study demonstrated that GQ and miR-451 exerted synergistic effect on reversing ADR-resistance in MCF-7/ADR cells by down-regulating the expression of MDR1 gene and the corresponding P-gp protein, indicating combination of chemical drug with miRNA is a promising strategy for reversing drug resistance in cancer therapy.

## MATERIALS AND METHODS

### Cell lines and cell culture

Human breast cancer cell line MCF-7 was purchased from Type Culture Collection of the Chinese Academy of Sciences (Shanghai, China). MCF-7/ADR (ADR-resistant) cell line was obtained from Pharmacy College of Dalian Medical University (Dalian, China). The cells were cultured in RPMI-1640 medium supplemented with 10% FBS (Thermo Fisher Scientific, Waltham, MA, USA) and 1% penicillin-streptomycin (Seven Biotech, Beijing, China) at 37° C under a humidified atmosphere with 5% CO_2_ [12].

### Animals

Four to six weeks old female BALB/c-nude mice were purchased from Liaoning Changsheng Biotechnology Co., LTD, China (SCXK: 2020-0001). Mice feeding and experiment were under a specific pathogen-free condition and in accordance with the Experimental Animal Management Law of China. Experiment protocol was approved by the Animal Ethics Committee of Dalian Medical University.

### Transfection of miRNA in MCF-7/ADR cells

ADR-resistant cells were seeded in 6-well plates (2×10^5^ cells per well) and incubated for 24 h until cell density reached 80~90%. MiRNAs and their inhibitors (GenePharma, Shanghai, China) displayed in Table 2 were mixed with cationic liposome CDO14 (synthesized in our lab) [34] at a weight ratio of 1:3 at 4° C for 20 min. The complexes (500 μL per well) were transfected into the cells and incubated for 6 h in serum-free RPMI-1640 medium. Then the cells were incubated in normal medium overnight and subjected to subsequent treatments.

**Table 2:** Sequence of miRNAs.

**miRNA**	**Sequence**
miR-451	Sense 5´-AAACCGUUACCAUUACUGAGUU -3´
	Antisense 3´-CUCAGUAAUGGUAACGGUUUUU -5´
miR-451 inhibitor	Sense 5´-AACUCAGUAAUGGUAACGGUUU -3´
miR-326	Sense 5´-CCUCUGGGCCCUUCCUCCAG -3´
	Antisense 3´-GGAGGAAGGGCCCAGAGGUU-5´
miR-326 inhibitor	Sense 5´-CUGGAGGAAGGGCCCAGAGG -3´
miR-328	Sense 5´-CUGGCCCUCUCUGCCCUUCCGU -3´
	Antisense 3´-GGAAGGGCAGAGAGGGCCAGUU -5´
miR-328 inhibitor	Sense 5´- ACGGAAGGGGAGAGAGGGCCAG -3´
miR-155	Sense 5´-AAACCGUUACCAUUACUGAGUU -3´
	Antisense 3´-CUCAGUAAUGGUAACGGUUUUU -5´
miR-155 inhibitor	Sense 5´- ACCCCUAUCACGAUUAGCAUUAA -3´
miR-NC	Sense 5´- UUCUCCGAACGUGUCACGUTT -3´
	Antisense 3´- ACGUGACACGUUCGGAGAATT -5´
miR-NC inhibitor	Sense 5´- CAGUAGUUUUGUGUAGUACAA -3´

### Cell viability assay

Cell counting kit-8 (CCK-8) assay was performed to measure cell viability for evaluating the role of miRNAs on ADR-resistance. ADR-resistant cells were cultured in 96-well plates at the initial density of 2×10^4^ cells per well for 18 h. MiR-NC, miR-326, 328, 451 and 155 and their inhibitors (1.5 μg, 500 μL) were respectively transfected into the cells for 6 h. MiRNAs-transfected or untransfected ADR-resistant cells were subsequently subjected to 43 μM ADR (Haizheng Pharmaceutical Co, Ltd, Zhejiang, China) for 48 h. Ten microliter of CCK-8 (Biotool, Shanghai, China) was added into each well and the cells were incubated for 2 h. A microplate reader (Thermo Fisher Scientific, Waltham, MA, USA) was used to detect absorbance at 450 nm.

### Determination of drug-resistant folds

CCK-8 assay was also used to determine half inhibition concentration (IC_50_) values of ADR to MCF-7 and ADR-resistant cells. The cells were seed in 96-well plates (2×10^4^ cells per well) and cultured for 24 h. MiR-326, 328, 451 and miR-155 inhibitor (1.5 μg, 500 μL) were respectively transfected into ADR-resistant cells for 6 h. The transfected and untransfected ADR-resistant cells, MCF-7 cells were administrated with ADR at various concentrations (decreasing proportionally from 172 μM) for 48 h. CCK-8 assay was performed as described above. IC_50_ values of ADR to MCF-7 cells, ADR-resistant cells, miRNAs-transfected ADR-resistant cells, and drug-resistant folds were calculated. Drug-resistant fold was the IC_50_ value of ADR against ADR-resistant cells (or miRNAs-transfected ADR-resistant cells) divided by that against MCF-7 cells. Reversal fold was the drug-resistant fold of ADR-resistant cells divided by that of miRNAs-transfected ADR-resistant cells.

Effect of GQ and miR-451 on ADR-resistance in MCF-7/ADR cells was detected. The cells were transfected with miR-451 (1.5 μg, 500 μL) for 6 h and then administrated with 10 μM of GQ (synthesized in our lab) [37] and ADR at various concentrations for 48 h. CCK-8 assay was used to detect cell viability and IC_50_ value was calculated.

### Detection of gene expression

Real-time RT-PCR assay was performed to detect the expression levels of miR-328, 326, 451, 155 in MCF-7 and ADR-resistant cells. The cells were incubated in 6-well plates at an initial density of 2×10^5^ cells per well for 24 h. Then cell lysis, RNA extraction and reverse transcription reaction were conducted as reported methods [12]. Real-time qPCR was performed according to the manufacturer’s recommendations and our previous study [12]. Endogenous U6 snRNA was used to normalize gene expression levels, the relative expression of miRNAs was quantified using 2^-ΔΔCt^ method [38].

Effect of miR-328, 326, 451, 155 and their inhibitors on the expression of MRP1, BCRP and MDR1 in ADR-resistant cells was detected by Real-time RT-PCR according to above methods [12]. β-actin mRNA was used to normalize the mRNA levels of MRP1, BCRP and P-gp.

Real-time RT-PCR was also performed to investigate the influence of GQ and miR-451 on the expression of MDR1 gene in ADR-resistant cells. The cells were seed in 6-well plates with a density of 2×10^5^ cells each well and incubated for 18 h. MiR-451 (1.5 μg, 500 μL) was transfected into the cells for 6 h. Both miR-451-transfected and untransfected ADR-resistant cells were administrated with ADR (43 μM) or ADR (43 μM) combining GQ (10 μM) for 48 h. The expression level of MDR1 gene in the cells was detected by Real-time RT-PCR as described above. The sequences of the above gene primers (GenePharma, Shanghai, China) were shown in Table 3.

**Table 3:** Sequence of PCR primers.

**Gene name**	**Primer sequence**
miR-451	Forward 5´-GCCGCAAACCGTTACCAT-5´
	Reverse 5´-TATCGTTGTTCTGCTCTCTGTCTC-3´
miR-326	Forward 5´-AGTGTCTCCTCTGGGCCCTT-3´
	Reverse 5´-TATGGTTGTTCACGACTCCTTCAC-3´
miR-328	Forward 5´-TGGTACTGCTGGCCCTCTCT-3´
	Reverse 5´-TATGGTTGTTCACGACTCCTTCAC-3´
miR-155	Forward 5´-GCTTCGGTTAATGCTAATCGTG-3´
	Reverse 5´-CAGAGCAGGGTCCGAGGTA-3´
miR-NC	Forward 5´-ACAGCAACUGGCUAUGGCdTdT-3´
	Reverse 5´- GCAUUGGGUUACAUUTT -3´
U6	Forward 5´-AGTGTCTCCTCTGGGCCCTT-3´
	Reverse 5´-TATGGTTGTTCACGACTCCTTCAC-3´
MDR1	Forward 5´-CCCATCATTGCAATAGCAGG-3´
	Reverse 5´-GTTCAAACTTCTGCTCCTGA-3´
BCRP	Forward 5´-ATCTTGGCTGTCATGGCTTCA-3´
	Reverse 5´-TCTTCGCCAGTACATGTTGCA-5´
MRP1	Forward 5´-ATGTCACGTGGAATACCAGC-3´
	Reverse 5´-GAAGACTGAACTCCCTTCCT-5´
β-actin	Forward 5´-GAGCCACATCGCTCAGACAC-3´
	Reverse 5´-CATGTAGTTGAGGTCAATGAAGG-5´

### Detection of protein expression

Western blot was performed to detect the effect of miRNAs on the expression of P-gp, BCRP and MDR1 in ADR-resistant cells. The cells (2×10^5^ cells per well) were cultured in 6-well plates overnight and subsequently transfected with miR-326, 328, 451, 155 and their inhibitors (1.5 μg, 500 μL) for 48 h. After that, the cells were collected and lysed as the described methods [36]. The protein samples were quantified using BCA assay kit (Beyotime, Shanghai, China) and stored at -80° C.

The influence of GQ and miR-451 on the expression of P-gp was also investigated by western blot. ADR-resistant cells were cultured in 6-well plates at the initial density of 2×10^5^ cells per well overnight following by transfection of miR-451 (1.5 μg, 500 μL) for 6 h. MiR-451-transfected and untransfected ADR-resistant cells were administrated with ADR (43 μM) or ADR (43 μM) combining GQ (10 μM) for 48 h. Then the cells were lysed and kept as described above. The protein samples were subjected to SDS-PAGE at 30 μg per lane, and then to western blot as reported methods with β-actin as an internal control [39].

### Scratch test

ADR-resistant cells were incubated in 6-well plates with the initial density of 2×10^5^ cells per well for 12 h. The cells in each well were divided on average with a UV-sterilized ruler and pipette tips, and washed twice to remove detached cells and debris. Some cells were transfected with miR-451 (1.5 μg, 500 μL) for 6 h before treatments of GQ (10 μM), ADR (43 μM) or their combination for 48 h while others subjected to the same treatments directly. Then the cells were photographed under an inverted microscope (GE, USA) to observe the width of scratches.

### Tumor inhibition in mice

The mixture of ADR-resistant cells (5×10^7^ cells/mL) and matrigel at equal volume was subcutaneously inoculated in the right armpit of female BALB/c nude mice (200 μL per mouse). Volume of the xenograft tumor was measured and calculated as follows: Volume = L×W^2^/2 (L and W respectively represented the length and width of the tumor) [12]. Twenty-four mice with tumor about 150 mm^3^ were divided into eight groups in random, and respectively treated with normal saline (Control), GQ, ADR, miR-451, ADR combining GQ, ADR combining miR-451, ADR combining GQ and miR-451, ADR combining GQ and miR-NC. The complex of liposome CDO14 and miR-451 (0.55 mg/kg) with the ratio of 3:1, ADR (3 mg/kg) and normal saline were injected via the tail vein, and GQ (18 mg/kg) was administered by gavage [39]. Tumor-bearing mice were administered once every three days for 6 times. Body weight, tumor volume and physical state were recorded daily until the mice were killed at 18th day. Tumors stripped from the mice were photographed and then subjected to western blot and IHC assay for detecting the expression of P-gp in tumor tissues.

A part of tissues was cut into small pieces and lysed in RIPA buffer to obtain protein for western blot assay according to reported methods [36]. Another part of tumor tissues were fixed in formalin fixative and penetrated in paraffin. Paraffin sections were made and IHC assay was performed as reported methods [12].

### Statistical analysis

Statistical analysis was performed using SPSS 21.0 (SPSS, Inc, Chicago, IL, USA). All data are presented as the mean ± standard deviation (SD) of at least three independent replicates. Student’s t-test was applied to assess the difference among groups. *P* < 0.05 was considered statistically significant.
